# Effect of Acetazolamide as Add-On Diuretic Therapy in Patients With Heart Failure: A Meta-Analysis

**DOI:** 10.7759/cureus.37792

**Published:** 2023-04-18

**Authors:** Bilal Aziz Malik, Ijeoma Nnodebe, Azrung Fayaz, Habiba Inayat, Syeda Fatima Murtaza, Muhammed Umer, Syed Asjad Tauheed Zaidi, Adil Amin

**Affiliations:** 1 Internal Medicine, CMH Lahore Medical College and Institute of Dentistry, Lahore, PAK; 2 Medicine, Basingstoke and North Hampshire Hospital, Basingstoke, GBR; 3 Medicine, Khyber Teaching Hospital, Peshawar, PAK; 4 Internal Medicine, Hayatabad Medical Complex Peshawar, Peshawar, PAK; 5 Medicine, Allama Iqbal Medical College, Lahore, PAK; 6 Internal Medicine, Dow University of Health Sciences, Civil Hospital Karachi, Karachi, PAK; 7 Medicine, Shalamar Medical and Dental College, Lahore, PAK; 8 Cardiology, Pakistan Navy Station (PNS) Shifa, Karachi, PAK

**Keywords:** all-cause mortality, meta-analysis, heart failure, diuretic, acetazolamide

## Abstract

The aim of this meta-analysis was to assess the effectiveness of acetazolamide as an add-on diuretic therapy in patients with heart failure. This meta-analysis was conducted in accordance with the PRISMA (Preferred Reporting Items for Systematic Reviews and Meta-Analysis) guidelines. A systematic literature search was independently performed by two authors using MEDLINE, EMBASE, and the Cochrane Database of Systematic Reviews to identify relevant studies assessing the use of acetazolamide in patients with heart failure. The search keywords included "acetazolamide" and "heart failure". The outcomes assessed in this meta-analysis included natriuresis (mmol/L), diuresis (Liters) and decongestion (absence of signs of volume overload) by 72 hours. Other outcomes assessed in this meta-analysis included hospitalization due to heart failure and all-cause mortality. A total of three studies included a total of 569 heart failure patients. The number of patients achieved decongestion was significantly higher in patients receiving acetazolamide compared to the patients randomized in the control group (RR: 1.34, 95% CI: 1.06-1.67). Compared to patients in the control group, mean natriuresis was significantly higher in acetazolamide patients (MD: 74.91, 95% CI: 39.85-109.97). Diuresis was significantly higher in patients receiving acetazolamide compared to the control group (MD: 0.44, 95% CI: 0.16-0.72). No significant difference was found between the two groups in terms of all-cause mortality and hospitalization due to heart failure. In conclusion, our meta-analysis suggests that acetazolamide may have beneficial impacts on heart failure patients by increasing the number of successful decongestions. Additionally, patients who were treated with acetazolamide had significantly higher natriuresis and diuresis compared to patients in the control group.

## Introduction and background

Heart failure affects 1 to 2% of people worldwide, with a higher prevalence in developed countries [1]. However, its economic burden has grown worldwide. It is also associated with an increased risk of premature death and hospitalization [2]. Fluid retention can lead to various symptoms in heart failure patients [3]. The typical method to address fluid retention in heart failure is by administering diuretics, but there is no evidence suggesting that this approach lowers mortality rates. Nonetheless, the European Society of Cardiology (ESC) suggests administering diuretics to achieve proper fluid balance in patients experiencing symptoms of overhydration, both in chronic and acute phases [4]. Although high-dose loop diuretics are utilized, a considerable number of patients are discharged from the hospital with lingering indications of fluid buildup, which is an influential predictor of unfavorable consequences [5-6]. As an illustration, research such as the Diuretic Optimization Strategies Evaluation (DOSE) study has demonstrated that just 15% of patients display no signs of clinical congestion even after being treated for 72 hours [7]. In addition, the Acute Decompensated Heart Failure National Registry (ADHERE) showed that around 20% of patients left the hospital with an increase in body weight [8]. Even though sequential diuretic therapy has been demonstrated as an efficient decongestive strategy compared to loop diuretics alone, evidence related to the efficient diuretic agent, routes of administration, and schedules of administration is limited [9].

Acetazolamide is a medication that inhibits carbonic anhydrase and prevents the proximal tubular absorption of sodium [10]. It was initially introduced as a diuretic for the treatment of congestive heart failure, as it was discovered to be more effective and less harmful than sulfanilamide diuretics. In the 1950s, a number of case studies involving the use of acetazolamide on heart failure patients showcased its successful decongestive properties [11-12]. Although loop diuretics are generally considered more potent, the utilization of acetazolamide has been decreasing [13]. Recently, new evidence has emerged indicating that the ceiling effect of acetazolamide results from a compensatory increase in distal tubular Na-Cl co-transporter activity due to a decline in pendrin expression [14]. This finding suggests that the combined use of acetazolamide and thiazide diuretics may be an effective treatment for diuretic resistance.

Acetazolamide decreases proximal tubular reabsorption of sodium and may improve diuretic efficiency when administered with diuretics, thus possibly facilitating decongestion [15]. However, there are limited human studies available testing the effectiveness of acetazolamide in the treatment of fluid overload in heart failure patients. Therefore, we conducted this meta-analysis to assess the effectiveness of acetazolamide as an add-on diuretic therapy in patients with heart failure.

## Review

Methodology

This meta-analysis was conducted in accordance with the PRISMA (Preferred Reporting Items for Systematic Reviews and Meta-Analysis) guidelines.

Search Strategy

A systematic literature search was independently performed by two authors using MEDLINE, EMBASE, and the Cochrane Database of Systematic Reviews to identify relevant studies assessing the use of acetazolamide in patients with heart failure from inception to 15th March 2023. The search keywords included "acetazolamide" and "heart failure" along with relevant medical subject heading (MeSH) terms and Boolean algebra operators to increase search sensitivity. In addition, the reference lists of all relevant articles were manually searched to identify additional articles relevant to the study objective.

Selection Criteria

We included observational studies or clinical trials that assessed the effect of acetazolamide therapy on decongestion, natriuresis, all-cause mortality, and hospitalization due to heart failure. Studies that lacked a comparison group, case reports, case series, and review articles were excluded. We also excluded studies published in languages other than English. All retrieved articles were independently reviewed by the two investigators using titles and abstracts. The full-text of all articles was obtained, and a detailed assessment was conducted to determine whether the articles were eligible to be part of the study or not. Any disagreements between the two authors in the study selection process were resolved by consensus.

Outcomes and Quality Assessment

The outcomes assessed in this meta-analysis included natriuresis (mmol/L), diuresis (Liters) and decongestion (absence of signs of volume overload) by 72 hours. Other outcomes assessed in this meta-analysis included hospitalization due to heart failure and all-cause mortality. Quality assessment of included randomized control trials (RCTs) was performed using the Cochrane Risk of Bias Assessment tool, which assessed the domains of selection, performance, attrition, reporting, and other biases. Quality assessment was performed independently by two authors, and any disagreements in the process of risk of bias assessment were resolved via discussion.

Data Extraction and Statistical Analysis

Data from included studies were extracted using a structured data collection form. The data extracted from all studies included the name of the first author, publication year, study design, study groups, acetazolamide regimen and dosages, sample size, patients' characteristics, and outcome measures.

"RevMan Version 5.4.1 (The Cochrane Collaboration, London, United Kingdom)" was used for the analysis. For categorical outcomes, the risk ratio (RR) was calculated with a 95% confidence interval (CI) using the Mantel-Haenszel approach. To estimate the effect of continuous measures, mean difference (MD) with a 95% CI was computed. Given the likelihood of between-study variance, we used a random-effects model rather than a fixed-effects model. I-square statistics and Cochran-Q test statistics were applied to assess the between-study heterogeneity. An I-square value ranging from 0% to 25% implies that the heterogeneity is not significant, while an I-square value ranging from 26% to 50% indicates low heterogeneity. If the I-square value falls between 51% and 75%, the heterogeneity is moderate, and an I-square value ranging from 76% to 100% indicates high heterogeneity.

Results

**Figure 1:**
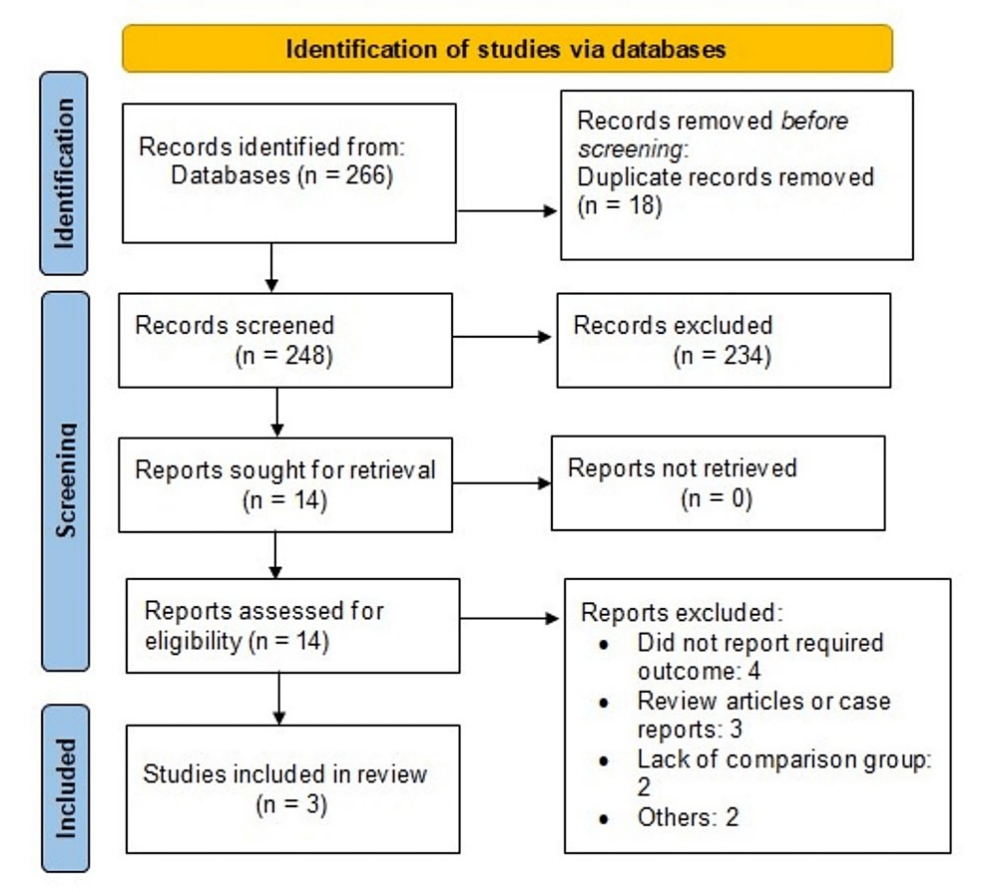
PRISMA flowchart of selection of studies

**Figure 2:**
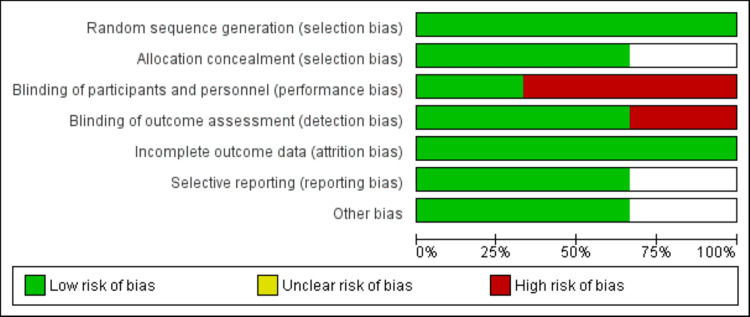
Risk of bias graph

A total of 266 eligible studies were identified through initial database searching. After removing duplicates, 248 records were assessed using the title and abstract. The full text of 14 articles was obtained, and a detailed assessment of the studies was conducted. Ten out of the final 14 records were excluded from the full-length review. As a result, the final analysis included studies consisting of three randomized control trials (RCTs) with a total of 569 heart failure patients. The process of literature retrieval, review, and selection is shown in Figure 1, and Table 1 displays the characteristics of the included studies. The studies were published between 2017 and 2022, and the majority of the participants were male. Figure 2 shows a summary of the risk of bias assessment of included studies.

**Table 1:** Characteristics of Included Studies RCT: Randomized control trial

Author Name	Year	Study Design	Participants	Groups	Dose	Sample Size	Age (Years)	Males (%)
Imiela and Budaj [14]	2017	RCT	Patients with chronic heart failure	Acetazolamide	250–375 mg daily	10	73 vs 71.2	80 vs 90
				Control		10		
Mullens et al. [15]	2022	RCT	Hospitalized adult patients with acute decompensated heart failure and at least one clinical indication of excess fluid volume.	Acetazolamide	500 mg daily	256	77.9 vs 78.5	65.6 vs 59.6
				Control		259		
Verbrugge et al. [16]	2019	RCT	Patients with clinical diagnosis of acute heart failure made within 8 hours. All patients demonstrated ≥2 clinical signs of congestion	Acetazolamide	250–500 mg daily	18	81 vs 78	61 vs 69
				Control		16		

Meta-analysis of outcomes

Decongestion, Natriuresis and Diuresis

**Figure 3:**
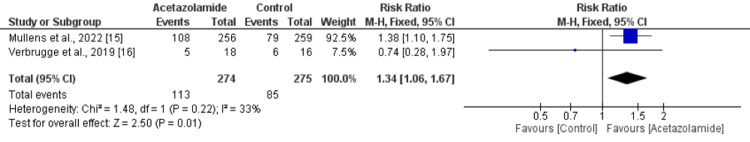
Effect of acetazolamide on decongestion Sources: References [15-16]

**Figure 4:**
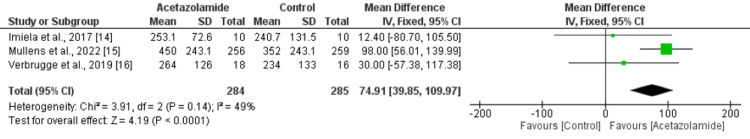
Effect of acetazolamide on natriuresis Sources: References [14-16]

**Figure 5:**
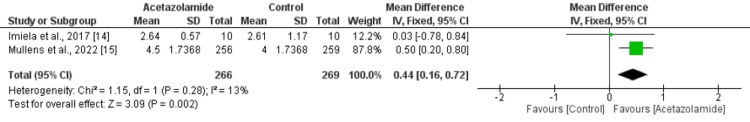
Effect of acetazolamide on diuresis Sources: References [14-15]

Two studies examined the number of patients who achieved decongestion within 72 hours of beginning treatment. The patients who received acetazolamide had a significantly higher number of patients who achieved decongestion than those in the control group (RR: 1.34, 95% CI: 1.06-1.67) according to Figure 3. There was low heterogeneity among the study results (I-square: 33%). Three studies evaluated natriuresis in a total of 569 patients. Compared to those in the control group, the acetazolamide patients had a significantly higher mean natriuresis (MD: 74.91, 95% CI: 39.85-109.97) as illustrated in Figure 4. Low heterogeneity was reported among the study results (I-square: 49%). Two studies compared diuresis between the two groups. The meta-analysis found that patients who received acetazolamide had significantly higher diuresis than the control group (MD: 0.44, 95% CI: 0.16-0.72) as shown in Figure 5. There was low heterogeneity among the study results (I-square: 13%).

All-cause Mortality and Heart Failure Hospitalization

**Figure 6:**
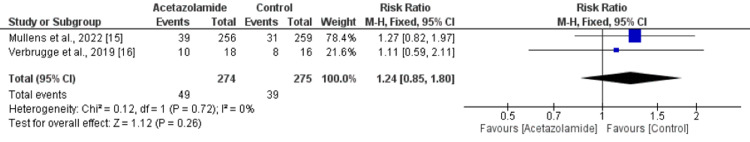
Effect of acetazolamide on all-cause mortality Sources: References [15-16]

**Figure 7:**
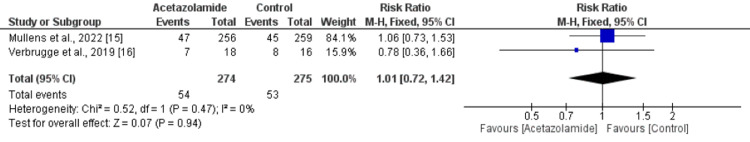
Effect of acetazolamide on hospitalization due to heart failure Sources: References [15-16]

Two studies assessed all-cause mortality and hospitalization due to heart failure between the two groups. No significant difference was found between the two groups in terms of all-cause mortality and hospitalization due to heart failure as shown in Figure 6 and Figure 7, respectively.

Discussion

In the present meta-analysis, we examined the impacts of using acetazolamide on patients with heart failure. The current meta-analysis reported that patients treated with acetazolamide had significantly higher natriuresis and diuresis compared to patients in the control group. Our meta-analysis also found that when acetazolamide was added to loop diuretic therapy, it led to higher and faster decongestion. However, the majority of the weight in our meta-analysis is carried by one multicenter RCT, which included a total of 515 patients [15]. The other two studies were also RCTs but included only 20 and 34 patients with heart failure. The risk of all-cause mortality and hospitalization due to heart failure was not significantly different between the two study groups.

The benefit of acetazolamide in the attainment of successful decongestion has a class I recommendation from the American and European diagnosis of heart failure treatment [9, 17]. Considering that congestion is associated with adverse outcomes in heart failure patients, the advantages of acetazolamide in this population are important. It is likely that the increased occurrences of decongestion observed during treatment with acetazolamide were caused by the immediate and continued increase in urine and sodium excretion that resulted from the use of the medication. These results emphasize the significance of addressing congestion promptly and with a strong approach, and they support the use of natriuresis as a way to measure the effectiveness of diuretic treatment [15].

Considering the pathophysiology involved, there are various reasons to use acetazolamide in combination with loop diuretic therapy to treat heart failure [18]. Firstly, acetazolamide increases the amount of sodium supplied to Henle's loop, which enhances the natriuretic effect of loop diuretics [19]. This meta-analysis confirms that loop diuretics work better in patients treated with acetazolamide, as evidenced by enhanced diuresis and natriuresis. It is important to note that the effectiveness of loop diuretics has been shown to be a strong and independent predictor of clinical outcomes in acute heart failure, although it has not been confirmed whether this relationship is causal [20]. Secondly, proximal renal sodium reabsorption inhibition leads to increased delivery of sodium and chloride to macula densa cells located at the end of the loop of Henle, which activates tubuloglomerular feedback in the same way as sodium-glucose co-transporter 2 (SGLT2) inhibitors do [21]. Lastly, acetazolamide may have inherent protective effects for the kidneys. In animal studies, acetazolamide has been shown to prevent ischemia-reperfusion injury, possibly by promoting vasodilation through the stimulation of nitric oxide [22]. Moreover, several studies predating the current era of heart failure treatment with neurohumoral blockers utilized acetazolamide as an efficient agent for overcoming resistance to loop diuretics [23-24]. This study extends these earlier findings to contemporary patients with advanced cardiorenal disease receiving modern treatment for acute heart failure and confirms that acetazolamide is a safe and effective medication to use in this context.

Study Limitations

There are several limitations to this meta-analysis. Firstly, only three studies were included in this meta-analysis. Most of the weight in the pooled analysis was carried by a single study [15]. Secondly, we were not able to perform subgroup analysis on the basis of certain groups like history of diabetes, kidney disease and others. Thirdly, we did not assess publication bias as not three studies were included in the present meta-analysis. Moreover, the dose of acetazolamide ranged from 250 to 500 mg in included studies. Due to a lack of individual patients' data and the limited number of studies, we were not able to conduct a subgroup analysis based on the dose of acetazolamide.

## Conclusions

In conclusion, our meta-analysis suggests that acetazolamide may have beneficial impacts on heart failure patients by increasing the number of successful decongestions. Additionally, patients who were treated with acetazolamide had significantly higher natriuresis and diuresis compared to patients in the control group. However, the current meta-analysis did not demonstrate any significant differences in all-cause mortality and hospitalization due to heart failure. Conducting additional studies to investigate the mechanisms, safety, and potential benefits of combining acetazolamide with other diuretics would be a crucial next stage before it can be widely adopted in clinical practice.
